# Modeling Prion-Like Processing of Tau Protein in Alzheimer’s Disease for Pharmaceutical Development

**DOI:** 10.3233/JAD-170727

**Published:** 2018-03-13

**Authors:** Claude M. Wischik, Björn O. Schelter, Damon J. Wischik, John M. D. Storey, Charles R. Harrington

**Affiliations:** a TauRx Therapeutics Ltd., Singapore; b School of Medicine, Medical Sciences and Nutrition, University of Aberdeen, Aberdeen, UK; c Institute for Complex Systems and Mathematical Biology, University of Aberdeen, Aberdeen, UK; d Computer Laboratory, University of Cambridge, Cambridge, UK; e Department of Chemistry, University of Aberdeen, Aberdeen, UK

**Keywords:** Alzheimer’s disease, clinical trials, paired helical filaments, prion-like processing, protein aggregation inhibitors, tau protein

## Abstract

Following our discovery of a fragment from the repeat domain of tau protein as a structural constituent of the PHF-core in Alzheimer’s disease (AD), we developed an assay that captured several key features of the aggregation process. Tau-tau binding through the core tau fragment could be blocked by the same diaminophenothiazines found to dissolve proteolytically stable PHFs isolated from AD brain. We found that the PHF-core tau fragment is inherently capable of auto-catalytic self-propagation *in vitro*, or “prion-like processing”, that has now been demonstrated for several neurodegenerative disorders. Here we review the findings that led to the first clinical trials to test tau aggregation inhibitor therapy in AD as a way to block this cascade. Although further trials are still needed, the results to date suggest that a treatment targeting the prion-like processing of tau protein may have a role in both prevention and treatment of AD.

## “PRION-LIKE” PROCESSING OF TAU PROTEIN

**Fig.1:**
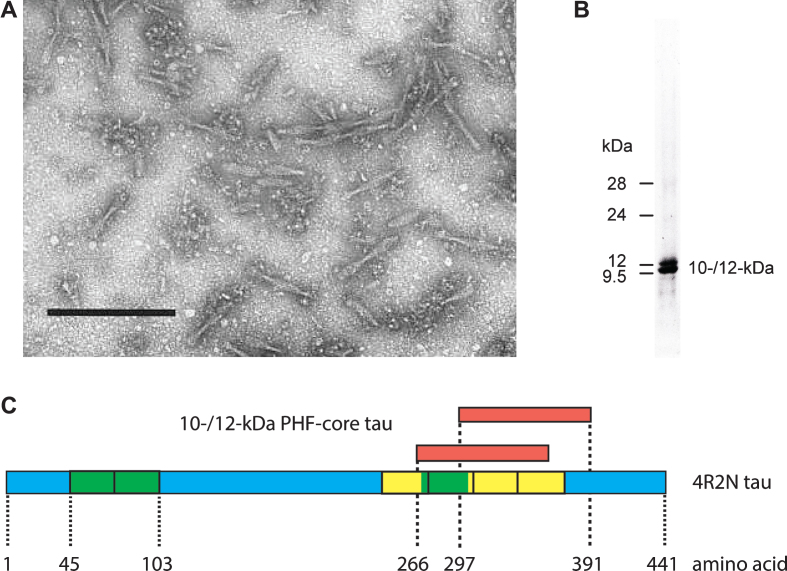
Tau composition of the PHF core. Fragmented PHFs (A), isolated from AD brain tissue, release a 10–12 kDa doublet following extraction with formic acid (B). This doublet corresponds to fragments from the C-terminal part of tau protein that contain the repeated tubulin-binding domain (yellow boxes) and vary depending upon the inclusion or exclusion of an additional repeat (green box) inserted in the protein by alternative splicing (C). Scale bar in A, 200 nm.

We first used the term “prion-like” in connection with the pathological processing of tau protein as a basis for developing a treatment in Alzheimer’s disease (AD) exactly 20 years ago [1]. This was the title of an extensive review summarizing our work over the preceding 12 years. Since then, the concept of prion-like processing of proteins to form characteristic pathological aggregates has come to be understood as a general molecular mechanism underlying a number of progressive neurodegenerative disorders [2–6]. For us, the idea arose from attempts to understand the surprising ability of a family of molecules of the diaminophenothiazine class to reverse the proteolytic stability of the otherwise highly resistant tau polymers, known as paired helical filaments (PHFs), isolated from the brains of patients dying with AD. We had found [7, 8] that it was possible to prepare an essentially pure preparation of PHFs from AD brain tissues using a combination of differential centrifugation and digestion with a broad spectrum exoprotease (Pronase). The resulting PHFs retained their typical structural characteristics apart from the loss of a proteolytically susceptible fuzzy outer coat [9, 10]. Treatment of these preparations with formic acid released a 10–12 kDa fragment of tau protein restricted to the repeat domain of the molecule [8], thereby establishing this fragment as a critical structural constituent of the proteolytically stable core of the PHF and not simply one of the many proteins which can be labelled in tangles and co-purify with PHFs in crude brain extracts [11–21](Fig. 1).

Since this fragment of tau accounted for 92% of the total protein content of such preparations, it followed that molecules of the diaminophenothiazine type must be blocking a tau-tau binding interaction that is critical for maintaining the structural and proteolytic stability of the PHF. Pre-treatment with a diaminophenothiazine eliminated the proteolytic stability of the 10–12 kDa protein fragment [22]. This implied that the proteolytic stability of the PHF core unit was not inherent to the tau molecule, but was rather secondary to its configuration within the assembled filament.

**Fig.2:**
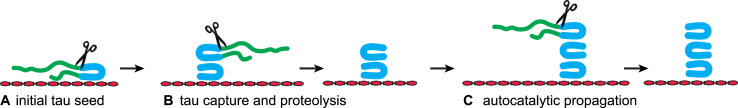
Tau aggregation *in vitro* demonstrates that the tau fragment from the PHF core permits autocatalytic propagation at the expense of normal tau. When incubated in the solid phase, the tau fragment from the core of the PHF binds full-length tau with high affinity and converts the repeat domain from the bound full-length tau molecule into a proteolytically stable replicate of the starting fragment. In these assays, the broad spectrum exoprotease Pronase was used [22]. A neo-epitope at the C-terminus of the newly truncated fragment recognized by mAb 6/423 (which recognizes a C-terminal truncation at position Glu-391) is created after digestion and is amplified in the course of repeated binding/digestion cycles. The core domain is shown in a C-shaped hairpin conformation to take account of what is now known of the atomic structure of this fragment within the PHF-core [39].

In order to understand the mechanism better, we developed a relatively simple *in vitro* tau-tau binding assay in which the PHF-core tau fragment (Ile-297 - Ala-390) was first incubated in the solid phase, and the binding of full-length recombinant 4-repeat tau added in the aqueous phase was measured immunochemically [22, 23] (Fig. 2). Three surprising observations emerged from these studies. The first was that binding could be blocked by the same diaminophenothiazines found to dissolve proteolytically stable PHFs isolated from AD brain [22]. The second was that after digestion of the bound complex with Pronase, full-length tau lost its N-terminal domain and acquired a neo-epitope detected with mAb 6/423 which recognizes a C-terminal truncation at Glu-391 present in the core of the PHF [22, 24]. Since both species in the bound complex lacked this epitope, its appearance after exoprotease digestion could only be explained as having come from full-length tau as the proteolytically stable footprint of the tau-tau binding interaction in the repeat domain. The third feature was that the binding interaction could be propagated through repeated binding/digestion cycles after addition of fresh full-length tau at each cycle with increasing accumulation of the proteolytically stable core unit in the solid phase [22]. Taken together, these observations implied that the tau-tau binding interaction occurring through the repeat domain of the molecule was responsible for both the proteolytic and structural stability of the of PHF and was inherently auto-catalytic and self-propagating, i.e., “prion-like”[1].

These properties were later confirmed in a stable cellular model system [25]. The core 10–12 kDa tau unit is highly toxic when overexpressed in cells. In order to model the templated truncation process within a cellular environment, it was necessary to establish a dual expression system in cells that do not normally express tau, namely fibroblasts. In this system, the PHF-core tau unit is expressed constitutively at very low non-toxic levels, and full-length four-repeat tau is expressed under the control of an inducible vector. Induction of full-length tau leads to its templated conversion to the core unit with the characteristic 10–12 kDa gel mobility. This conversion was blocked quantitatively by the same diaminophenothiazine-class molecules which had been found to dissolve proteolytically stable PHFs and which also blocked the tau-tau binding assay modelled in a cell-free environment [25]. Therefore, once present, the core-tau unit is able to propagate itself at the expense of normal full-length tau within the normal cellular milieu.

**Fig.3:**
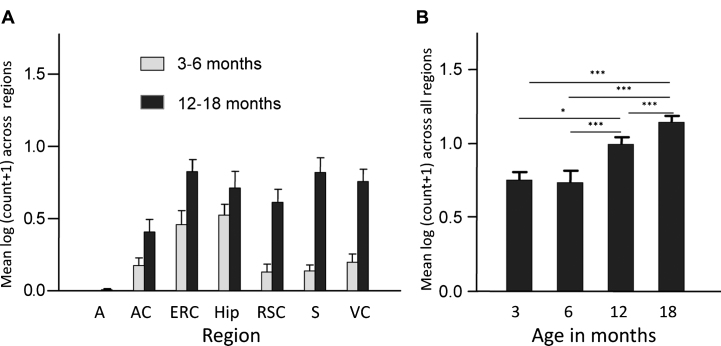
Transgenic tau mice exhibiting both regional and age-related spread of tau immunoreactivity. Line 1 mice demonstrate a significant age-related spread of tau-reactive cells from entorhinal cortex and hippocampus to neocortex that is reminiscent of Braak staging, and a corresponding increase in numbers of cells having tau pathology. A, amygdala; AC, auditory cortex; ERC, entorhinal cortex; Hip, hippocampus; RSC, retrosplenial cortex; S, subiculum; VC, visual cortex. Source: From Melis et al., *Cell Mol Life Sci* 72, 2199-2222, 2015.

Finally, overexpression of the PHF-core tau unit in a transgenic mouse model produced tau aggregation pathology which is restricted to entorhinal cortex and hippocampus in 3-6-month-old mice, and spreads to isocortex only after mice are 12 months old (Fig. 3). This pattern of spread reproduces a characteristic feature of the tau pathology of AD that forms the basis of the Braak staging system in humans [27] discussed further below. A significant cognitive deficit was present already from 3 months of age in the absence of any motor impairment. Amelioration of both tau aggregation pathology and cognitive impairment were achieved following six weeks treatment with methylthioninium (MT) [26], the prototype diaminophenothiazine we have taken into clinical development and which is discussed further below.

## STRUCTURE AND ASSEMBLY OF PHFS

**Fig.4:**
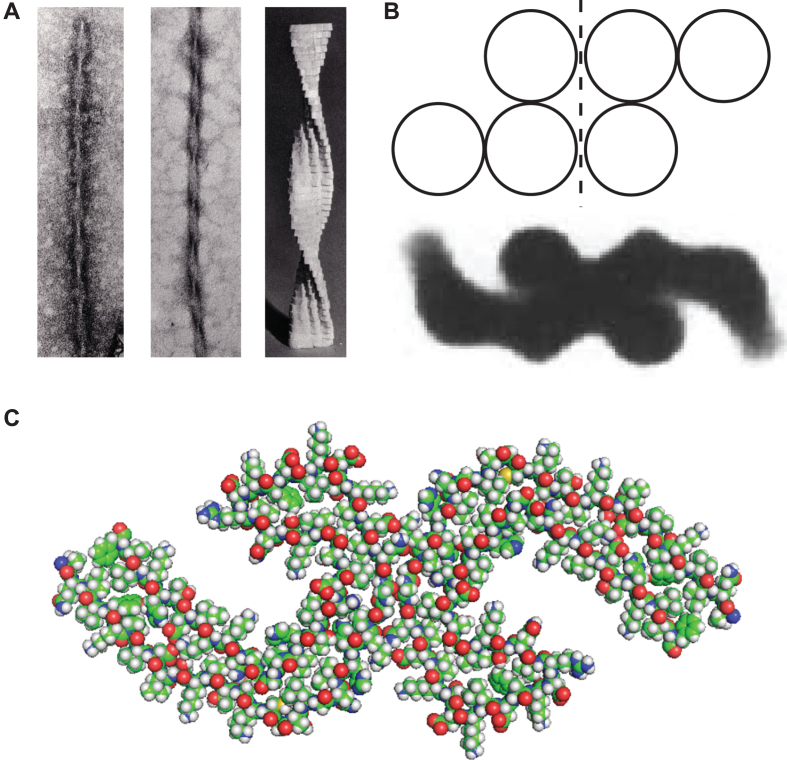
The subunit structure of the PHF core. (A) PHFs, isolated from AD brain tissue, are surrounded by a fuzzy coat (left panel) that is removed by proteolysis to reveal a PHF having a protease-resistant core and characteristic filamentous appearance (middle panel). Model reconstruction (right panel) revealed a C-shaped subunit structure in transverse section (B), consistent with the atomic structure which has been determined by cryo-electronmicroscopy of PHFs (C) [39].

PHFs isolated from AD brain tissue without Pronase digestion have a fuzzy outer coat (Fig. 4A). Although such PHFs are still recognized by mAb 6/423, they require higher concentrations of the antibody to do so [24]. This suggests that the N- and C-terminally truncated repeat domain tau unit is present within PHFs in a manner that is partially occluded by the fuzzy coat. This interpretation was supported by immunohistochemical studies examining the sequencing of tau aggregation and truncation in AD brain tissues. The aggregates recognized by mAb 6/423 are initially amorphous and are not labelled by benzothiazole fluorophores that bind to assembled PHFs. As these aggregates are converted to PHFs, they acquire the ability to bind benzothiazoles, but lose mAb 6/423 immunoreactivity. The latter can be revealed by formic acid treatment of the histological section [28].

The fuzzy coat contains the portions of the tau molecule that are N-terminal to the repeat domain core. Immunoreactivity associated with the N-terminal portion of the molecule is lost after proteolytic removal of the fuzzy coat [8, 10]. Since the core structure remains intact after removal of the fuzzy coat, it can be concluded that the N-terminal half of the tau molecule makes no contribution to the core, which contains no post-translational modifications [29]. The epitopes recognized by phosphate-dependent antibodies are located exclusively in the fuzzy coat [30].

These complex immunochemical properties have led to considerable confusion in the literature regarding the potential role of phosphorylation of tau protein both as a therapeutic target and as providing a conjectural bridge to altered process of AβPP in AD. A widely quoted misconception that has remained in the literature since 1991 [31] is that “PHFs are composed almost entirely of hyperphosphorylated tau protein”. However, the molecular mass of the PHF core is ∼65 kDa/nm [10], or ∼6.5×10 kDa core tau units per nm. Since the mass of the N-terminal half of the tau molecule is ∼23 kDa, the predicted mass of PHFs isolated without protease treatment would be ∼210 kDa/nm if all of the PHF tau protein were N-terminally intact. However, such PHFs have a maximal mass of 110 kDa/nm, and more typically 80–95 kDa/nm [10]. This implies that the bulk of the molecular mass of the PHF is made up of truncated tau, and only 1 in 7 tau molecules is N-terminally intact. These approximations, based on structural data, agree well with direct immunochemical measurements of PHF preparations, since phosphorylated tau accounts for less than 5% of total PHF-tau measured independently of phosphate-dependent immunoreactivity [7, 32]. Whether full-length hyperphosphorylated tau protein is simply attached on the outside of the core polymer, or whether some of the structural tau units of the core are N-terminally intact is not known at present. Either way, it is extremely unlikely that PHFs could be composed “almost entirely of hyperphosphorylated tau protein” as widely supposed.

Systematic tau-tau binding studies *in vitro* using the assay described above showed that, as the concentration of either truncated tau in the solid phase or full-length tau in the aqueous phase species increases, their respective binding affinities increase to asymptotic values of 21.1±2.9 and 31.5±22.6 nM, respectively [33]. This implies that a conformational change can be induced in a concentration-dependent manner which facilitates tau-tau binding. This concentration-dependent enhancement is not seen in the tau-tubulin binding interaction, which has a 19-fold lower binding affinity of 403±86 nM irrespective of tau or tubulin concentration [33].

Both tau-tau and tau-tubulin binding through the repeat domain are profoundly disturbed by phosphorylation. In the case of tau-tau binding, phosphorylation produces a 10- to 50-fold inhibition depending on the precise configuration of the measurement [33]. For tau-tubulin binding, the inhibition is 24-fold. Therefore, phosphorylation controls the folding of the tau molecule in solution in such a way as to occlude the availability of the binding domain. These findings also imply that it is unnecessary to invoke phosphorylation as a mechanism responsible for the transfer of tau protein from predominantly tubulin-bound to predominantly aggregated states, as is widely assumed [34]. Once the repeat-domain fragment is available in a suitable conformation, the dynamics of microtubule assembly and disassembly will favor the redistribution of the tau protein pool to the aggregated state simply by virtue of differential binding affinities. The progressive accumulation of endogenously truncated tau in AD brain suggests that the cellular clearance mechanisms are compromised once the repeat domain is locked in its proteolytically stable configuration and that the aggregated truncated form represents a kinetic sink [32]. Indeed, quantitative analyses of soluble and aggregated tau in AD brain tissues implied that the loss of soluble tau is insufficient to explain the accumulation of aggregated tau. Rather, it appears that the soluble tau pool is being maintained by increased production to compensate for loss into the aggregated form [32].

**Fig.5:**
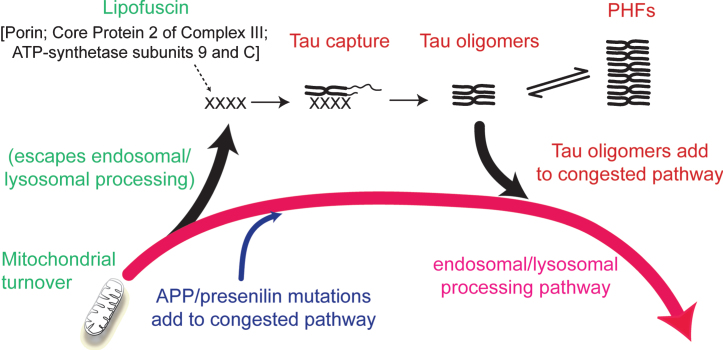
Involvement of the endosomal–lysosomal pathway in removal of aggregated proteins. Congestion of the clearance pathway associated with progressive age-related failure of normal mitochondrial turnover leads to release of products of failed clearance which become seeds for triggering tau aggregation. The resulting tau oligomers add to congestion in the pathway and themselves catalyze further tau aggregation. The tau aggregation cascade proceeds by an autocatalytic process of binding and proteolysis of tau, initiated through its capture of by-products of failed mitochondrial clearance resulting from age-related failure of endosomal–lysosomal processing. Source: From Wischik et al., *Biochem Pharmacol* 88, 529–539, 2014.

The inhibitory effect of phosphorylation can be eliminated by first binding full-length tau to the solid phase [33]. This removes the conformational protection afforded by phosphorylation which shields the repeat domain from pathological aggregation. There is some evidence supporting the idea that the critical binding substrate which enables the initial conformational change required to trigger the tau aggregation cascade may be related to products of incomplete mitochondrial clearance [1, 35]. Lipofuscin deposits which typically accumulate in aging neurons are composed of undigested products of mitochondrial turnover [36]. These mitochondria-derived fragments co-localize with truncated tau in lysosomes, co-purify with proteolytically stable PHFs and form SDS-resistant complexes with truncated tau [22, 35]. Although such aggregates may provide the primary substrate needed to seed tau aggregation, the process is self-propagating and autocatalytic once initiated. Such a scenario would then locate the initiation of tau aggregation within the framework of a more general age-related impairment in the efficiency of endosomal-lysosomal processing which is required for clearance of membrane-bound proteins (such as AβPP) and mitochondria [37, 38] (Fig. 5).

A low resolution structure of the PHF core was initially described on the basis of direct negative-stain electron microscopy observations and model building [9]. Isolated PHFs are characterized by longitudinal strands which transition between a 4-stranded to a 3-stranded appearance in the course of a left-hand helical rotation of an underlying ribbon-like structure (Fig. 4A). Since the filaments have clean transverse breaks, the underlying subunit must be transverse in orientation. These features can be explained by two C-shaped subunits back to back with a core unit consisting of three distinct domains. This structure was confirmed by image diffraction analysis of isolated core-PHFs (Fig. 4B).

More recently, this core structure has been confirmed more elegantly by cryo-electronmicroscopy which has permitted detailed resolution at the atomic level [39]. The sequence of the repeating core tau unit, determined structurally [39], corresponds almost exactly to the sequence first isolated from the PHF core 29 years ago [8]. The only difference is that the proteolytically stable unit, shown biochemically [8, 24], extends by 8 residues N-terminal and 13 residues C-terminal relative to the sequence that could be visualised by cryo-electronmicroscopy (i.e., Ile-297 –Glu-391, rather than Val-306 –Phe-378). The atomic resolution structure has made it possible to see for the first time that this core tau unit is arranged as a bent hairpin structure arranged to produce the C-shaped structural subunit (Fig. 4C). We have recently found that the core tau expressed *in vitro* unit exists in two distinct conformations with gel mobilities of 10- and 12-kDa, respectively [40]. Only the 12-kDa form is assembly competent, and assembles spontaneously into double helical ribbons with the same 4-stranded to 3-stranded transitions that indicate the presence of the same C-shaped subunit originally seen in PHFs. This suggests that the bent hairpin configuration of the repeat domain can occur in solution, that the bonds maintaining it are SDS-resistant even in the monomeric form, and that this critical conformational transition can occur in the absence of any post-translational modification [40]. In particular, these studies exclude any requirement for formation of disulphide cross-links in PHF assembly.

These structural findings have important implications for understanding exactly how diaminophenothiazines are able to disassemble the PHF core and reverse the proteolytic stability of the repeat domain in its assembly-competent configuration [22]. It has been known for some time that the tau-tau and tau-tubulin binding interactions are pharmacologically distinct, even though both occur through the repeat domain [22]. Using MT as the prototype compound, dissolution of PHFs isolated from AD brain can be demonstrated at MT concentrations approximately 1/10th that of the PHFs [25]. This makes it unlikely that the action of MT is competitive, but rather suggests a catalytic interaction of some kind. It is tempting to speculate that MT works by de-stabilizing the hairpin configuration of the of the core tau unit which makes it assembly-competent. This would result in reversion of the core tau unit into a form which is both assembly-incompetent and more susceptible to proteolytic pathways available for protein clearance [41]. In the cell model described above, which is an inherently dynamic system, the levels of the core tau unit relative to full-length tau are reduced after treatment with diaminophenothiazines, implying both inhibition of tau aggregation and enhanced clearance of tau aggregates. That is, the core tau unit is no longer kinetically trapped in the aggregated state in the presence of diaminophenothiazines. Interestingly, MT enhances autophagy and clearance of aggregated tau protein in a transgenic mouse model at 0.05μM [42] and, at low dose, induces genes regulated by NF-E2-related factor 2 (Nrf2) which control pathways available for clearance of proteotoxic proteins in another transgenic tauopathy mouse model [43]. How these effects are related to dissolution of tau aggregates is unknown, but both point to enhancement of the ability of neurons to clear them.

## POTENTIAL CLINICAL EFFICACY OF TAU PROTEIN AGGREGATION INHIBITOR THERAPY IN AD

**Fig.6:**
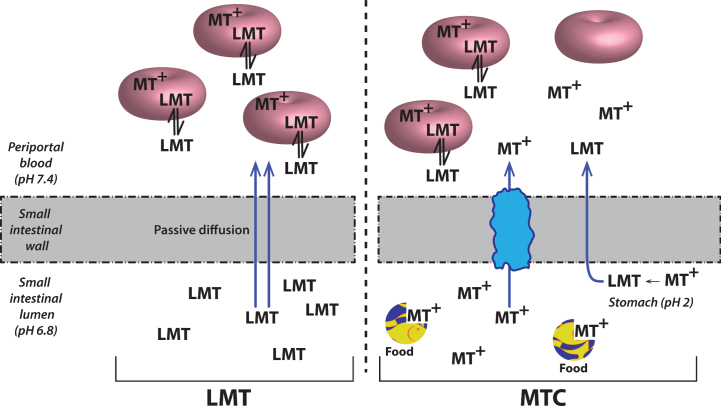
Cellular uptake of methylthioninium species. A model to explain the complex absorption characteristics of MT leading to differential disposition of LMT and MTC. Source: From Baddeley et al., *J Pharmacol Exp Ther* 352, 110-118, 2015.

Although MT has attractive properties *in vitro* and in transgenic mouse models as a possible treatment for the tau pathology of AD, its clinical pharmacology is complex. This is due in part to the redox lability of the molecule at physiological pH values. The best studied example is in the treatment of methemoglobinemia which is the only approved clinical indication for the use of MT. When the MT moiety is administered intravenously in its oxidized form (MT^+^) as methylthioninium chloride (MTC, commonly known as “methylene blue”) it needs first to be reduced to its uncharged form (LMT for “leuco-MT”) to permit it to cross the red cell membrane [44] (Fig. 6). It is then concentrated inside the cell as a result of an equilibrium between the oxidized form which is favored at physiological pH and the reduced form which is maintained in an energy-dependent fashion by consumption of reduced glutathione [44]. This dynamic equilibrium permits MT to transfer electrons to oxidized hemoglobin thereby reducing it. A similar electron shuttling mechanism is thought to underlie the ability of MT to enhance mitochondrial metabolism [45]. It is not known whether this redox lability is critical either for dissolution of PHFs or preventing tau aggregation.

The first tau protein aggregation inhibitor (TAI) to enter clinical development was MT in its oxidized MT^+^ form as MTC. As indicated above, MTC has a long history of safe use, both as an approved treatment for methemoglobinemia [46], and experimentally in urolithiasis and bipolar disorder amongst others [47–49]. A phase II clinical trial was conducted between 2004–2008 in 321 subjects with mild/moderate AD [50] The trial was designed as an exploratory double-blind, randomized, placebo-controlled, 24-week dose-finding study of MT as monotherapy in AD to test the doses 69, 138 and 218 mg day given in divided doses three times per day on clinical and functional brain imaging (HMPAO-SPECT) outcomes.

The primary efficacy analysis at 24 weeks showed that treatment with MT 138 mg/day is the minimum effective dose required to prevent disease progression when given as MTC. In the pre-specified primary efficacy analysis, treatment with 138 mg/day produced statistically significant benefit with respect to placebo on the ADAS-cog scale (effect size = –5.42 units, CI = [–9.44, –1.41], nominal *p* = 0.008, corrected *p* = 0.047) at 24 weeks in patients with moderate AD at baseline. Significant effects were also seen on the secondary outcome scales Alzheimer’s Disease Cooperative Study - Clinical Global Impression of Change (ADCS-CGIC) and Mini-Mental State Exam (MMSE) (effect size = 3.79 units, CI = [1.16, 6.41], nominal *p* = 0.0048, corrected *p* = 0.028). A significant treatment effect was also seen at the same dose on the functional molecular imaging outcome (HMPAO-SPECT) in a separate population (mild subjects) at 24 weeks (effect size = 1.97%, CI = [1.02, 2.92], nominal *p* < 0.001, corrected *p* < 0.001). Over 50 weeks, clinical benefit was seen in both mild and moderate AD (effect size = –3.80 Alzheimer’s Disease Assessment Scale–cognitive (ADAS-cog) units, CI = [–3.31, –3.25], nominal *p* = 0.004, corrected *p* = 0.011).

**Fig.7:**
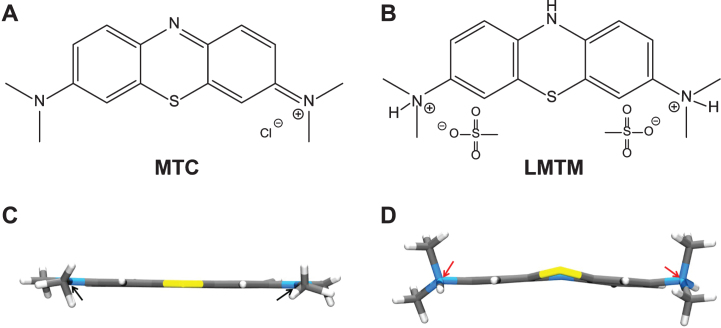
Structures of the diaminophenothiazines MTC and LMTM. Methylthioninium is a diaminophenothiazine which exists in a redox equilibrium between oxidized and reduced forms, represented here as MTC and LMTM (A, B), respectively. The X-ray crystal structures of the molecules shown below each form are distinct from each other (C, D); the nitrogen atoms are planar in the case of MTC and tetrahedral for LMTM (red arrows). The geometry of the nitrogen atoms contributes to the stability of LMTM in an oxygen atmosphere in its crystalline form as the dihydromesylate salt.

Surprisingly, a higher dose of 228 mg-MT/day was less effective on both clinical and functional imaging outcomes [50]. This was found to be due to dose-dependent impairment in absorption in the presence of food [51]. As indicated above, the oxidized MT^+^ needs to be reduced to the uncharged LMT form to permit absorption. This was confirmed *in vivo* by the observation that the red cell uptake of the LMT form is 20-fold better than the MT^+^ form [51]. Red cell uptake is important for distribution of MT to the brain, since this sequestration protects the molecule from an efficient first-pass metabolism which inactivates MT as a tau aggregation inhibitor [51]. The conversion from the MT^+^ form to the LMT form, which is favored at low pH values [52], most likely occurs in the stomach and is impaired in the presence of food. The further clinical development of the MT moiety in Phase 3 was therefore switched to a stable reduced dosage form as leuco-methylthioninium bis(hydromethanesulfonate) (LMTM). LMTM is a distinct novel chemical entity which retains tau aggregation inhibitor activity *in vitro* and *in vivo* [25, 26], has superior pharmaceutic properties in terms of solubility and pKa, and is not subject to the absorption limitations of the MT^+^ form as MTC [51](Fig. 7).

LMTM was tested in two Phase 3 randomized controlled double-blind clinical trials in AD. The first (TRx-237-015, “Study 015”) was a 15-month study in 891 patients with mild to moderate AD and compared 150 mg/day and 250 mg/day with a low dose intended as a control (8 mg/day) in divided doses twice per day [53]. The second (TRx-237-005, “Study 005”) was an 18-month study in 800 patients with mild AD and compared 200 mg/day with the same intended control in divided doses twice per day. The control dose of 8 mg/day was selected as the lowest dose found to be able to mask urine discoloration relative to higher doses in earlier Phase 1 studies, and was expected to be ineffective on the basis of the results of the earlier Phase 2 study using MTC. In addition to having an active control arm, the Phase 3 studies also differed from the earlier Phase II study in permitting LMTM to be taken as add-on to approved anti-dementia treatments (cholinesterase inhibitors and/or memantine), whereas the Phase 2 study did not.

In Study 015, it was shown that there was no difference between either of the two higher doses and the intended control dose on either of the co-primary outcomes (ADAS-cog and ADCS-ADL) or any of the secondary outcomes [53]. However, the primary prespecified analysis model showed that patients who received LMTM as monotherapy had a lower rate of progression (ADAS-cog, *p* < 0.0001; ADCS-ADL, *p* = 0.0174; ADCS-CGIC, *p* < 0.0001; MMSE, *p* < 0.0001). A further prespecified *post hoc* analysis was therefore undertaken in the whole population which included anti-dementia treatment status as an interaction term with LMTM treatment and as an interaction term with visit in the model. In patients taking LMTM as monotherapy, the differences with respect to the control arm were significant after correction for multiple comparisons on all treatment outcomes. For ADAS-cog, the effect size at 150 mg/day was –6.25 units (CI = [–8.92, –3.59], nominal *p* < 0.001) and for 250 mg/day was –5.79 units (CI = [–8.47, –3.11], nominal *p* < 0.001). For ADCS-ADL, the effect size at 150 mg/day was 6.48 units (CI = [2.87, 10.09], nominal *p* = 0.001) and for 250 mg/day was 6.93 units (CI = [3.29, 10.57], nominal *p* < 0.001). Similar results were seen for other clinical outcomes, and importantly the rate of brain atrophy was significantly less in the monotherapy patients. In patients taking the same doses of LMTM as add-on to approved anti-dementia treatments, the decline was indistinguishable from controls as randomized or from placebo controls in recently reported studies [54, 55].

When the control arm was analyzed in similar manner, patients receiving LMTM 8 mg/day as monotherapy also had significantly better outcomes than those receiving the same dose as add-on to approved anti-dementia treatments. For ADAS-cog, the difference between 8 mg/day as monotherapy relative to add-on was –5.90 units (CI = [–8.13, –3.66], nominal *p* < 0.001). For ADCS-ADL, the corresponding difference was 7.19 units (CI = [4.19, 10.23], nominal *p* < 0.001).

**Fig.8:**
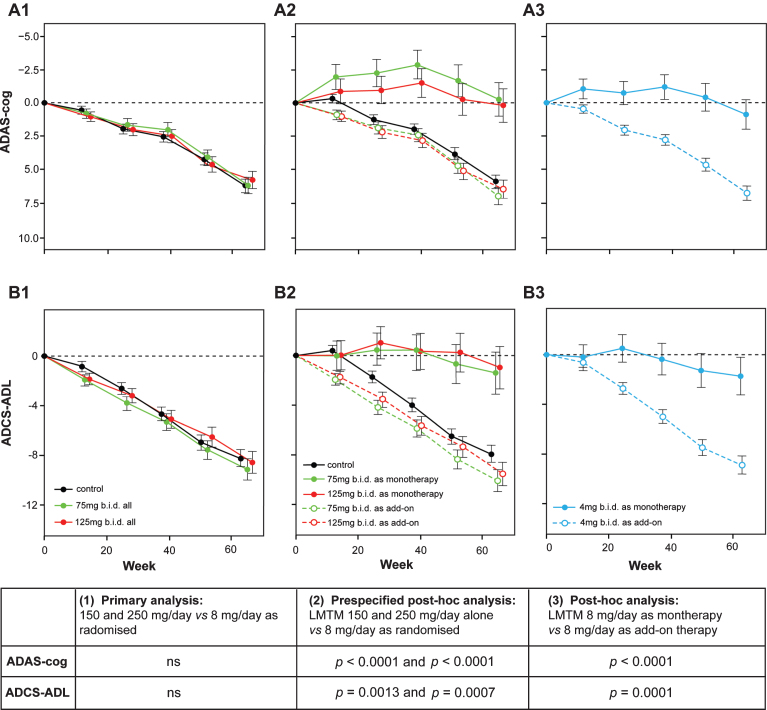
Change from baseline in ADAS-cog and ADCS-ADL for Study 015. The results are shown for the as-randomized primary analysis with AD co-medication status as an additive term in the model (A1, B1), or prespecified repeat of primary analysis with AD-co-medication status as an interaction term in the model showing effect of LMTM treatment as either monotherapy or as add-on to existing AD treatments (A2, B2). A *post-hoc* analysis is shown for low dose (8 mg/day) with LMTM given as monotherapy or as add-on (A3, B3). ns, not significant. Source: Adapted from Gauthier et al., *Lancet* 388, 2873-2884, 2016, with permission of Elsevier.

These differences between monotherapy and add-on could not be explained by differences in age or sex distribution, in baseline ADAS-cog or MMSE scores, or time between diagnosis and randomization. Patients with mild (but not moderate) AD who were not taking these medications were marginally worse on the ADCS-ADL scale, had a slightly greater hippocampal volume and smaller lateral ventricular volume on baseline MRI, and had a lower *APOE* *ɛ*4 allele carrier frequency. There were no differences in mild or moderate patients in whole brain volume, temporoparietal volume, or in the extent of vascular pathology burden. There was also no difference in the initial rate of expansion of lateral ventricular volume over the first 6 months. When the differences in baseline severity, hippocampal volume, and other parameters were corrected for, the differences in favor of monotherapy remained statistically significant (Fig. 8).

The results of Study 015, which became available prior to database lock and unblinding of Study 005, raised the possibility that LMTM might be effective only as monotherapy and that the minimum effective dose might be substantially lower for LMTM than that previously identified using MTC [50]. Since the originally intended analyses as randomized were unlikely to be able to achieve their intended purpose, the primary analyses and treatment comparisons in the statistical analysis plan for Study 005 were modified prior to database lock and unblinding to investigate whether the monotherapy differences could be confirmed as observational cohort comparisons defined as primary outcomes with strong control of family-wise type I error in the second independent study. The monotherapy cohort comparisons which were of particular interest in light of the earlier study were: 100 mg twice a day as monotherapy compared with 4 mg twice a day as originally randomized, and 4 mg twice a day as monotherapy compared with 4 mg twice a day as add-on to standard AD treatments. These were defined as two parallel primary comparisons which each had to be significant at *α*= 0.025 for the analysis to be successful. The results of Study 005 have been reported recently [56]. As expected, there was no evidence of any difference on any of the primary or secondary endpoints in the as-randomized analyses comparing all patients receiving LMTM at a dose of 100 mg twice a day and those receiving 4 mg twice a day. In the non-randomized cohort comparisons defined as the primary outcomes in the revised statistical analysis plan, both primary comparisons were statistically significant for both co-primary clinical outcomes (ADAS-cog and ADCS-ADL), as well as for volumetric MRI and glucose uptake (FDG-PET) biomarker outcomes. Patients receiving LMTM as monotherapy at either of the two doses tested had consistently better outcomes than patients receiving the same doses as add-on to anti-dementia treatments. These effects remained significant after correction for any potential effects of differences at baseline in patients taking or not taking approved anti-dementia treatments.

The confirmation of the same pattern of results in the second independent study argues against either set of findings being the result of chance in small subgroups, although the monotherapy subgroups remain small (15% and 20% in Studies 015 and 005, respectively). It is also unlikely that the earlier findings are explicable by inclusion of non-western geographies, since the second study was conducted in north America, western Europe, and Australia. A clinical placebo effect in patients coming into a trial setting after previously not receiving active treatment cannot explain the same pattern of results seen in both the MRI brain atrophy and glucose uptake data as seen in the clinical data. A difference in withdrawal rates between patients taking or not taking standard AD treatments is also unlikely, since the overall retention rates over 18 months were similar in monotherapy (65%) and add-on (69%) treatment groups in Study 005. The pattern of atrophy determined by MRI at baseline in patients receiving LMTM as monotherapy was typical of mild AD and significantly different from a cohort of well characterized normal elderlycontrols [57]. The annualized rate of whole brain atrophy in these patients over the first 6 months was also similar to that reported for mild AD and significantly different from normal elderly controls [58]. Likewise glucose uptake in inferior temporal gyrus was comparable in both monotherapy and add-on patients to that reported for mild AD [59] and significantly different from MCI or normal elderly controls [59]. There is no reason, therefore, to assume that patients not treated with approved AD treatments were anything other than typical mild AD patients. When the analyses were corrected for potential differences in baseline severity and a range of other baseline parameters, the results again remained robustly significant.

The potential for LMTM to be active at the low dose of 8 mg/day and the lack of dose-response was unexpected given the results of an earlier Phase II placebo-controlled study using MTC. However, as indicated, LMTM is now known to have a 20-fold better red cell uptake than MTC *in vivo* [51] and also better brain uptake [26]. A population pharmacokinetic analysis using blood samples collected in the course of the second study indicate that the higher dose produced higher plasma levels of MT. Using data from rat and pig to estimate brain levels from the plasma data obtained in the trial, the estimated brain concentration of MT at the 8 mg/day dose was in the range 0.05–0.2μM and 2.0–3.8μM at the 100 mg twice a day dose. The brain concentration estimated at the 8 mg/day dose is similar to the minimum effective concentration estimated from the earlier Phase II clinical trial using MTC [50, 51]. Although this difference in dosage requirement between LMTM and MTC is consistent with the 20-fold difference in cell uptake, this was not known at the time the Phase III studies were designed and initiated. The lack of dose-response may well be explained by a similar lack of dose-response for oligomer disaggregation *in vitro*, and higher doses of LMTM do not result in greater reduction in tau pathology in transgenic mouse models [26]. This suggests that there may be a critical threshold for the catalytic action of MT in the dissolution of PHFs and oligomers, and the effect of higher doses on pathology may plateau or may even become negative at brain concentrations above 1μM [26]. A similar inverse dose-response was also found in a different transgenic model of tau aggregation pathology in which mice received MTC in the drinking water from 1–9 months of age [43].

The negative interaction with symptomatic anti-dementia treatments is more difficult to explain. We have recently found that the interference is not all-or-none, but depends in part on relative basal forebrain atrophy. Our current working hypothesis is that long-term inhibition of cholinesterase activity (or indirect enhancement of cholinergic activity by memantine) combined with loss of inhibitory modulation from the basal forebrain may result in chronic hyperactivation of pyramidal cells in cortex which are the principal sites of neurofibrillary degeneration in AD [60]. We hypothesize that hyperactivation of cortical pyramidal cells may impair the action of MT even at high dose. It is unknown whether interference of this type is a feature of any treatment aiming to enhance clearance of aggregated tau, or whether it is specific to LMTM.

As a clinical summary, therefore, the same pattern of results has been seen now in two separate Phase III studies implying that the effects are consistent across studies. They are also internally consistent across a range of clinical and biomarker outcomes. Allowing for the differences in absorption between LMTM and MTC which are now known, the results are also consistent with the earlier Phase II placebo-controlled study supporting potential efficacy of the MT moiety as monotherapy. We believe that the within- and between-study consistency of the results point to clinical and biological effects of LMTM as monotherapy at the safe and well-tolerated dose of 8 mg/day twice a day which could provide a clinically meaningful addition to the available treatment options for AD. This possibility now needs to be confirmed in a further suitably randomized clinical trial in patients not currently receiving the approved anti-dementia treatments. Although the apparent treatment differences in favor of monotherapy do not appear to be the result of measurable differences between the cohorts, it is impossible to exclude unmeasured confounding effects without a further randomized trial comparing 8 mg/day against true placebo.

## LIFE-COURSE AND PREVALENCE OF TAU PROTEIN AGGREGATION

The potential for availability in the near future of a safe tau aggregation inhibitor therapy as monotherapy needs to be considered in the context of the life-course and prevalence of tau aggregation pathology. Numerous clinico-pathological studies have demonstrated correlations between tau pathology and the extent of clinical dementia [61–70]. The staging system for spread of tau aggregation pathology proposed by Braak and Braak [27] has proved a valuable tool in refining these correlations and understanding the prevalence of the process.

**Fig.9:**
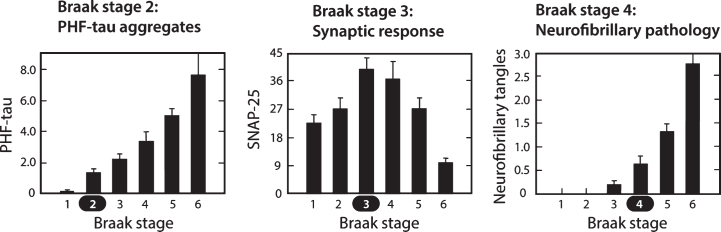
Molecular changes in neocortex by Braak staging. Proteolytically stable PHFs are present in neocortex from Braak stage 2 onwards (A); levels of SNAP-25 (as well as synaptophysin and syntaxin, not shown) increase significantly at Braak stage 3 and decline only after Braak stage 5 (B); significant numbers of neurofibrillary tangles are not observed until Braak stage 4 and beyond (C). Measures determined for frontal and temporal cortices. Source: Adapted from Mukaetova-Ladinska et al., *Am J Pathol* 157, 623-636, 2000, with permission of Elsevier.

The pattern of spread of the tau aggregation pathology in the human brain is highly characteristic and stereotyped. Layer II of entorhinal cortex is the first area to be affected in cortex, although there may be earlier involvement in basal forebrain and brainstem structures [2, 71]. We reported a study in a prospectively characterized neuropathological cohort in which we examined the sequence of changes in tau aggregation measured biochemically (as proteolytically stable PHF-tau levels) and corresponding changes in synaptic markers occurring in neocortex [68]. The Braak staging “clock” can be defined, at the early stages, on the basis of the pattern of spread of histopathological changes restricted largely to entorhinal cortex and hippocampus (stages 1–3). The sequence of changes in parietal and frontal neocortex, which begin later in terms of histopathology (stages 4–6), can then be analyzed at the molecular level as they develop against an initially clear histopathological background, using the Braak staging clock to order the sequence. These are summarized in Fig. 9.

In contrast to tau histopathology (measured as tangle counts) which becomes apparent in neocortex only from Braak stage 4 onwards, accumulation of proteolytically stable PHFs in the neocortex is already significant from Braak stage 2. In the neocortex, therefore, tangles are a relatively late manifestation of tau aggregation pathology. The calculated interval between onset of tau aggregation in neocortex and tangle histopathology is about 35 years. Braak stages 4 –6 represent the more typically recognized stages of pathology, in which the appearance of tau tangles and tau-positive, neuritic plaques occurs at approximately the same time. Braak stage 4 is a point of neuropathological transition that is expressed simultaneously as neurofibrillary tangles and neuritic/amyloid plaques. Further progression in Braak stages 5 and 6 is seen only for the tau and synaptic markers.

It is surprising to see that the changes in levels of synaptic proteins measured biochemically in the same brain regions follow a biphasic course, with an initial increase at Braak stage 3, followed by progressive decrease from Braak stage 4 onwards [68]. This implies that the appearance of measurable levels of proteolytically stable aggregated tau in the neocortex at Braak stage 2 is followed by a statistically significant increase in synaptic proteins at Braak stage 3. It is not known whether this increase is a compensatory consequence of a functional impairment in pyramidal transmission, or whether it represents a release phenomenon resulting in loss of inhibition resulting from basal forebrain atrophy where tau aggregation pathology is known to occur early in the sequence. Either way, the increase in synaptic markers suggests hyperactivation which may be accentuated by chronic treatment with cholinesterase inhibitors and memantine, as discussed earlier.

**Fig.10:**
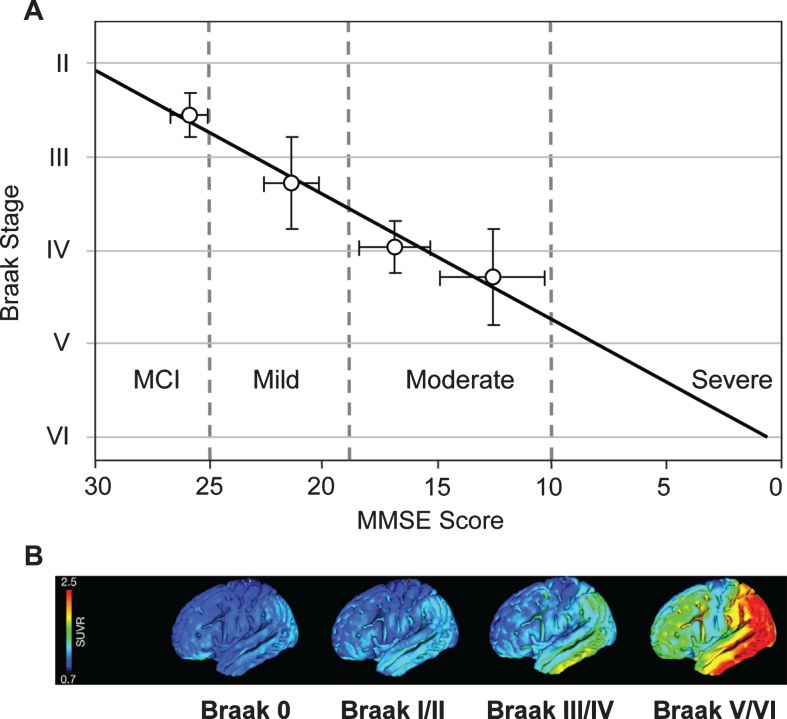
Correlation of Braak staging with cognitive decline and pathology. Cognitive decline is measured by MMSE determined 12–24 months antemortem and tau pathology measured *in vivo* using ^18^F-AV-1451 (tau) PET. Source: (A) From Wischik et al., *Biochem Pharmacol* 88, 529-539, 2014; (B) From Schöll et al., *Neuron* 89, 971-982, 2016, with permission of Elsevier.

Cognitive impairment in this epidemiological cohort was studied using MMSE scores obtained 12–24 months prior to death. From this, it was possible to derive an approximate trajectory linking Braak stage and MMSE score [68] (Fig. 10A). What is now referred to interchangeably as prodromal AD or mild cognitive impairment (i.e., MMSE score >25) occurs roughly between Braak stages 2 and 3. There are similar strong relationships between Braak stage and functional molecular imaging deficits as shown either by HMPAO-SPECT or FDG-PET which have similar ability to demonstrate deficits due to neuropathology [72, 73]. Both HMPAO-SPECT [74, 75] and FDG-PET [76] are correlated with Braak stage (Fig. 10B). The emergence of tau imaging ligands has made possible the direct imaging of tau aggregation pathology *in vivo* [77, 78]. This has permitted an explicit confirmation of the relationship between aggregation of tau and functional molecular imaging deficits. Functional deficit is strongly correlated with tau aggregation as shown by tau-PET imaging, but not with aggregation of amyloid-β as shown by imaging with^11^C-PIB [79, 80].

**Fig.11:**
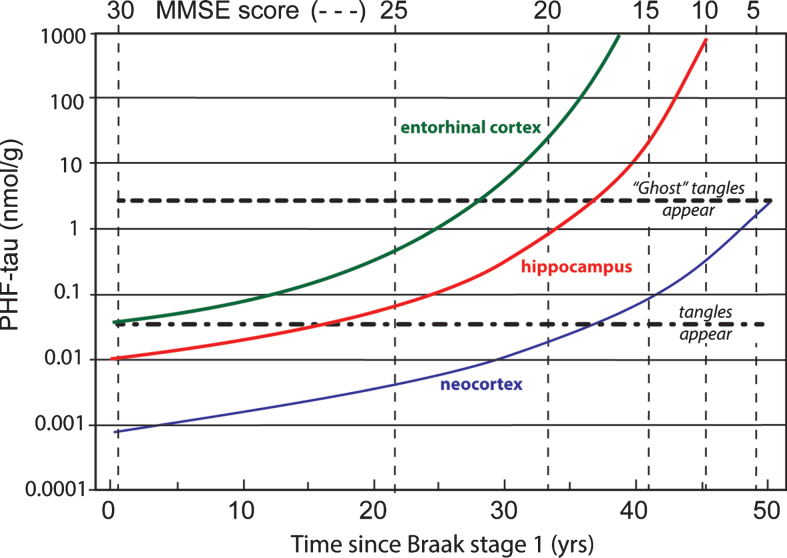
Time course of accumulation of aggregated PHF-tau in AD and associated cognitive decline on the MMSE scale.

Data coming from several clinico-pathological correlation studies are summarized in Fig. 11 showing the evolution of tau aggregation over time in archicortex and neocortex and the relationship to cognitive decline [7, 68, 81]. As can be seen, the entire sequence occurs over a 50-year time-span. The primary vertical axis shows levels of aggregated tau in the form of proteolytically stable PHFs measured in entorhinal cortex, hippocampus, and neocortex on a logarithmic scale. The progressive accumulation of aggregated tau is exponential over time, consistent with the autocatalytic characteristics of tau aggregation through the repeat domain discussed earlier. There is no evidence of abrupt transition between tau aggregation in medial temporal lobe structures and neocortex, except that aggregation in neocortex lags aggregation in entorhinal cortex andhippocampus.

**Fig.12:**
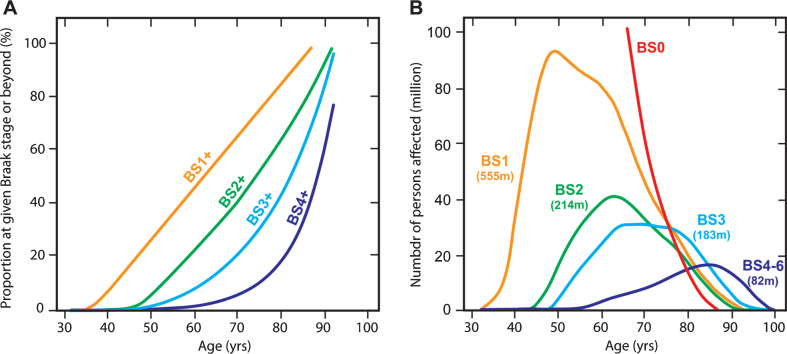
Age-associated probability of transition from an earlier to a later Braak stage (“BS”) (A) applied to estimate number of persons (in millions) worldwide at a given Braak stage in 2015 and their predicted age distribution (B).

An extremely useful data set from Ohm et al. [82] makes it possible to estimate population prevalence of tau aggregation pathology by Braak stage in Caucasian populations. We have applied the age-dependent Braak stage transition probabilities derived from this data set (Fig. 12A) to estimate the number of affected persons worldwide in 2015 using WHO data (Fig. 12B). For this purpose, we assume that the Ohm et al. data might be applicable also in Asian populations, although there is at present no evidence to support this, except that age-related prevalence of dementia appears to be similar [83]. We calculate that for the population over the age of 45, there is a 50% probability of having some degree of tau pathology in the brain. This can be divided as follows: 25% at Braak stage 1, 10% at Braak stage 2, 10% at Braak stage 3, and 5% at Braak stage 4 or beyond. We estimate therefore that there may well be almost 500 million people worldwide with tau aggregation at Braak stage 2 or beyond in their brains. The transition to Braak stage 2 and beyond is associated with significant impairment that can be measured even on the crude MMSE scale (Fig. 10). Of the people we estimate to be affected, only 12% would be in Europe or North America. Asia would account for an estimated 60% of the affected population. There is therefore an urgent need to develop an effective oral treatment for tau aggregation pathology that is convenient, safe and well-tolerated. If the results seen in two Phase 3 trials are confirmed in a further placebo-controlled trial, they point to the potential for LMTM to fulfilthis need.

## DISCLOSURE STATEMENT

Authors’ disclosures available online (https://www.j-alz.com/manuscript-disclosures/17-0727r1).
